# Tiludronate and clodronate do not affect bone structure or remodeling kinetics over a 60 day randomized trial

**DOI:** 10.1186/s12917-018-1423-2

**Published:** 2018-03-20

**Authors:** Heather A. Richbourg, Colin F. Mitchell, Ashley N. Gillett, Margaret A. McNulty

**Affiliations:** 1 Department of Orthopaedic Surgery, University of California San Francisco, San Francisco, CA USA; 2 Department of Comparative Biomedical Sciences, Louisiana State University School of Veterinary Medicine, Baton Rouge, LA USA; 3 Department of Veterinary Clinical Sciences, Louisiana State University School of Veterinary Medicine, Baton Rouge, LA 70803 USA; 4 Department of Anatomy and Cell Biology, Indiana University School of Medicine, Indianapolis, IN USA

**Keywords:** Horse, Bone, Bisphosphonate, Clodronate, Tiludronate, Micro-computed tomography, Histomorphometry, Biopsy

## Abstract

**Background:**

Tiludronate and clodronate are FDA-approved bisphosphonate drug therapies for navicular disease in horses. Although clinical studies have determined their ability to reduce lameness associated with skeletal disorders in horses, data regarding the effect on bone structure and remodeling is lacking. Additionally, due to off-label use of these drugs in young performance horses, effects on bone in young horses need to be investigated. Therefore, the purpose of this randomized, experimental pilot study was to determine the effect of tiludronate and clodronate on normal bone cells, structure and remodeling after 60 days in clinically normal, young horses. Additionally, the effect of clodronate on bone healing 60 days after an induced defect was investigated.

**Results:**

All horses tolerated surgery well, with no post-surgery lameness and all acquired biopsies being adequate for analyses. Overall, tiludronate and clodronate did not significantly alter any bone structure or remodeling parameters, as evaluated by microCT and dynamic histomorphometry. Tiludronate did not extensively impact bone formation or resorption parameters as evaluated by static histomorphometry. Similarly, clodronate did not affect bone formation or resorption after 60 days. Sixty days post-defect, healing was minimally affected by clodronate.

**Conclusions:**

Tiludronate and clodronate do not appear to significantly impact bone tissue on a structural or cellular level using standard dose and administration schedules.

## Background

Tiludronate disodium (Tildren, Ceva Animal Health LLC, Lenexa, KS, USA) and Clodronate disodium (Osphos, Dechra, Ltd., Staffordshire, UK) are bisphosphonate drugs that are licensed for use in horses to reduce lameness associated with navicular disease [1, 2]. Tiludronate and clodronate are non-nitrogen containing bisphosphonates that reduce osteoclastic bone resorption by causing osteoclast apoptosis [3]. Tiludronate has been used to treat conditions associated with bone remodeling, such as navicular disease [4, 5] and tarsal osteoarthritis [6]; however, none of these studies have evaluated the effect of tiludronate on bony tissue and have only evaluated the effects on lameness outcomes. Clodronate is similar to tiludronate in drug properties and has been shown to have an analgesic effect by acting on glutamate and/or adenosine triphosphate-related pain transmission pathways [7]. Tiludronate has not been shown to have such analgesic properties. The Freedom of Information (FOI) Summary for both clodronate and tiludronate report on the clinical outcome in a group of horses diagnosed with navicular disease. Treated horses displayed clinical improvements in their degree of lameness; however, neither FOI Summary study discerns whether the reduction in lameness associated with either drug is due to the effect on bone remodeling, analgesic potential (specifically in regards to clodronate), or other mechanisms.

The FOI summary for clodronate describes the effects on bone mineral content, cortical bone strength and bone marrow evaluation evaluated by radiographic photometry, mechanical testing and histopathology, respectively, in normal horses. No difference were reported in these parameters between treated and saline controls at 6 months [1]. However, the methods regarding how bone density data were obtained were not stated, and given the poor sensitivity of radiographs to determine bone density [8], it is uncertain whether there was or was not a true effect on bone in this cohort. The FOI summary for tiludronate evaluated the 3rd metatarsal and metacarpal, 3rd carpal and navicular bone in 30 horses for pathological bone lesions using histopathology and found no abnormal bone tissue or resorption sites [2]. However, given the little details provided on how these assessments were performed, these outcomes may not be sensitive or reliable enough to determine bony effects.

Despite its known effect on bone remodeling rates in other species, the effect of clodronate on bone healing, especially in the horse, is unclear. In animal models, clodronate has been found to not alter endochondral bone formation within the fracture callus or epiphyseal plate of rats [9, 10]. However, reports regarding effects of clodronate are conflicting, including no changes in bone mineral density in a callus [11], 30% increase in bone mineral density [10], increased calcium content within the callus [12], and decreased healing callus strength [13]. Recently, it has been shown that osteoclasts are a necessary component of efficient endochondral ossification during fracture repair in mice [14]; therefore, it is possible that clodronate may impair normal bone healing in horses, which calls for careful consideration when used in a clinical setting.

The gold standard for bone analyses are histomorphometry [15] and micro-computed tomography (microCT) [16], both of which require bone biopsies. The tuber coxae has been reported to be the easiest site for bone biopsy acquisition in the horse [17, 18] and is a region that was found to have consistently higher levels of tiludronate in comparison to other bones [19]. A study published by the authors has shown the tuber coxae as a site to obtain reliable bony samples in a non-terminal equine model that are consistent in size and of adequate quality to evaluate trabecular bone using both histomorphometry and microCT [17].

Therefore, the purpose of this study is to evaluate the effect of clodronate and tiludronate on bone morphology, bony cells and bone remodeling in young horses. Additionally, the effect of clodronate on bone healing after an induced defect was also investigated. These evaluations were performed by obtaining bone biopsies of the tuber coxae using an established technique [17], and evaluated through histomorphometry and microCT. To evaluate bone healing, subsequent biopsies were taken from the initial biopsy site, which served as a novel bone defect model in the horse. We hypothesized that tiludronate and clodronate would reduce osteoclast number and function, resulting in increased bone volume and increased bone apposition when compared to baseline biopsies and untreated horses, as well as increased bone formation after injury in clodronate treated horses.

## Methods

### Study design

The experimental protocol was approved by the Louisiana State University Institutional Animal Care and Use Committee (IACUC). Nineteen Thoroughbred horses, between 2 and 5 years of age were obtained via donation to the Louisiana State University Equine Health Studies Program Herd. Medical history of the horses was unknown at the time of donation; however, all horses underwent at least a two-week isolation and washout period following donation prior to enrollment in the current study. Horses were randomly assigned, via a coin flip, to either a treatment (TIL: *n* = 5, CLO: n = 5) or control group (SAL: *n* = 9) (Table 1). Two separate studies were completed, individually assessing the impact of TIL or CLO respectively, resulting in slightly different study designs (i.e., inclusion of the re-biopsy outlined below in the CLO study). No significant differences in control groups were found, therefore they were combined into a single control group. Because this is a novel pilot study, sample sizes were calculated based on available data for tiludronate in another species using the same endpoints (microCT & histomorphometry) [20], which found that *n* = 5 was sufficient to identify bony changes. Additionally, horses served as their own control, as bilateral biopsies were taken from each animal, thereby eliminating the need for additional horses. The surgeon, who was not involved in subsequent analyses, was not masked to treatment groups.

**Table 1:** Age (years), treatment and biopsies collected from study subjects. Day 0 biopsies were baseline biopsies collected on Day 0, Day 60 biopsies were collected from the contralateral side 60 days post-treatment, and Day 60R biopsies were collected from the ipsilateral side 60 days post- defect and post-treatment. Summary Information for Study Subjects

ID	Age (yrs)	Treatment	Biopsies collected
1	5	Saline	Day 0, Day 60, Day 60R
2	4	Saline	Day 0, Day 60, Day 60R
3	3	Saline	Day 0, Day 60, Day 60R
4	4	Saline	Day 0, Day 60, Day 60R
5	3	Saline	Day 0, Day 60
6	5	Saline	Day 0, Day 60
7	2	Saline	Day 0, Day 60
8	3	Saline	Day 0, Day 60
9	4	Saline	Day 0, Day 60
10	5	Tiludronate	Day 0, Day 60
11	3	Tiludronate	Day 0, Day 60
12	3	Tiludronate	Day 0, Day 60
13	5	Tiludronate	Day 0, Day 60
14	2	Tiludronate	Day 0, Day 60
15	5	Clodronate	Day 0, Day 60, Day 60R
16	4	Clodronate	Day 0, Day 60, Day 60R
17	4	Clodronate	Day 0, Day 60, Day 60R
18	3	Clodronate	Day 0, Day 60, Day 60R
19	3	Clodronate	Day 0, Day 60, Day 60R

**Fig. 1:**
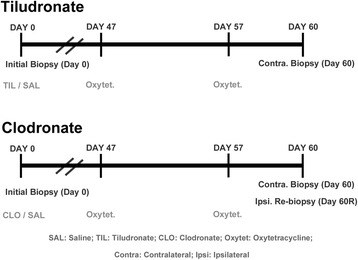
Study design. Study design time line, outlining biopsy collection and treatment administration

Bone biopsies were taken from each subject; a baseline biopsy at Day 0, a contralateral biopsy 60 days post-treatment (Day 60) (Fig. 1). Additionally, a re-biopsy of the initial biopsy site was taken from clodronate-treated horses and a subset of control horses 60 days post-treatment (Day 60R). This 60-day time frame was determined based on data in previous studies that showed a positive effect of tiludronate administration on various skeletal disorders in horses 60 days post initiation of treatment [5, 6, 21]. The right or left tuber coxae was randomly selected, via coin flip, for the first biopsy (outlined below in “Surgical Procedure”). Immediately following the initial biopsy surgery (Day 0), each horse was either administered 1 L of 0.9% saline IV (tiludronate control), 1 mg/kg of tiludronate dissolved in 1 L of 0.9% saline IV, or 1.8 mg/kg of clodronate IM (dose was divided into three injection locations) or a similar volume of 0.9% saline IM (clodronate control). Saline and tiludronate were infused intravenously over 90 min using an IV fluid pump. The horses stood in the stocks during drug administration and were monitored for signs of colic. Sixty days later, the contralateral tuber coxae was biopsied in the same manner. Additionally, clodronate treated horses (*n* = 5) and half of the control horses (*n* = 4) were evaluated for bone healing after a defect. Therefore, an additional biopsy (60R Day) was obtained from these horses only by taking a re-biopsy of the initial biopsy site, 60 days post- defect (i.e., the original biopsy collection). Prior to the 60 day biopsies, oxytetracycline (Vetrimycin 100, VetOne, Boise, ID, USA) was administered at Day 47 and Day 57 as a fluorochrome label. Biopsies were evaluated with microCT and histomorphometry for changes in bone morphology and remodeling rates, as outlined below under “Biopsy handling and imaging”.

### Study subjects

Thoroughbred horses, as outlined above under “Study Design”, were included if they were free from outward musculoskeletal disease as assessed by a boarded veterinary surgeon during a physical exam, and were between two to five years of age. A coin flip was used to randomly assign horses to a treatment group (e.g., treated or saline) and only the surgeon (CFM) knew group assignments until subsequent statistical analyses on data collected were ready to be performed. Throughout the study, they were housed in individual stalls following the biopsies and then turned out in groups in pastures. They were fed free choice hay and water, with grain being provided twice daily.

### Surgical Procedure & Treatment

Horses were restrained in stocks and sedated with IV xylazine (Xylamed, Bimeda, Cambridge, ON, Canada) (0.35 to 0.5 mg/kg). The surgical method has been previously published in detail [17]. In short, the biopsy site was centered on the proximal palpable protuberance of the tuber coxae. A rectangular region (approximately 10 cm X 10 cm) was clipped around the tuber coxae and then aseptically prepared. Lidocaine (Lidocaine 2%, VetOne, Boise, ID, USA) was injected subcutaneously 4 cm proximal and distal to the palpable tuber coxae, and then deeply to the periosteum of the tuber coxae. The horse was further sedated with detomidine (Dormosedan, Zoetis, Kalamazoo, MI, USA) (3 to 5 mg IV) and butorphanol tartrate (Torbugesic, Fort Dodge, New York, NY, USA) (3 to 5 mg IV) prior to making the skin incision [17].

A vertical incision was made over the tuber coxae, and dissected to expose the cranial, caudal, proximal and axial margins. The lateral periosteum was incised using a scalpel blade, and then an oscillating saw was used to transect the proximal portion of the tuber coxae. In order to limit thermal damage, the saw blade was continuously lavaged with saline. The surgery site was lavaged, and then the subcutaneous tissue and skin were sutured separately. An aluminium based bandage spray (Aluspray, Neogen Corporation, Lexington, KY, USA) was applied to the surgery site and a lidocaine patch (Lidocaine Patch 5%, Qualitest, Huntsville, AL, USA) was then applied over the incision [17].

Following the initial biopsy (Day 0 Biopsy), horses were stalled for at least 24 h and monitored every 2 h over the first 24-h period using a visual pain score [22]. After 24 h, horses were returned to their respective pastures and checked daily for signs of lameness or incisional complications until suture removal at 14 days post biopsy. At Day 47 and 57 post-biopsy, the horses were treated with 25 mg/kg of oxytetracycline administered slowly IV [17, 23]. Sixty days after the initial biopsy, the contralateral tuber coxae was biopsied (Day 60 biopsy), and the ipsilateral tuber coxae was re-biopsies in a subset of horses (Day 60R biopsy) using the same surgical methods as described above. Careful consideration was made when placing the oscillating saw for the re-biopsy (60R) to ensure an adequately-sized biopsy of the tuber coxae was obtained without cutting into the pelvis. In addition to aforementioned post-surgical care for the initial biopsy, after surgery to obtain the 60R biopsy, horses were administered flunixin meglumine (Prevail, Bimeda, Cambridge, ON, Canada) (1.1 mg/kg IV) for an additional analgesic. Surgical aftercare was then identical following this repeat biopsy to the care following the initial biopsy from the post-surgery period to 14 days later when the sutures were removed.

### MicroCT

Following biopsy, tuber coxae samples were dissected free from tissue and fixed in 10% non-buffered formalin for 7 days, then transferred to and stored in 70% ethanol at 20 °C. Samples were placed in holders with 70% ethanol for scanning by microCT (Scanco model 40, Scanco Medical AG, Basserdorf, Switzerland). Biopsies were scanned at 55 kV, 0.3-s integration time, with a 30 μm voxel size in plane and a 30 μm slice thickness. MicroCT data for two Day 0 biopsies from clodronate-treated samples were not obtained because motion artifact occurred during the scan, and this issue was not identified until the samples had been decalcified for histology. The region of interest was determined for trabecular bone. The proper threshold for image segmentation was tested, with the same threshold being used throughout the experiment to ensure consistency and accuracy in measurements between samples. Trabecular bone was evaluated for bone volume (BV), total volume (TV), BV/TV, tissue mineral density, trabecular number (TbN), trabecular thickness (TbTh), trabecular separation (TbSp), connectivity density (ConnD), and structural model index (SMI) based on established procedures and nomenclature [24]. A detailed list of these parameters, including definitions, has been previously published by Bouxsein, et al. (Table 2 within reference [24]).

**Table 2:** Summary data (mean ± SD) of bone structure and remodeling parameters from Day 0, Day 60 and Day 60R biopsies for micro-computed tomography (MicroCT), static and dynamic histomorphometry (histo) analyses. Groups are separated based on treatment received

Saline	MicroCT				Static histomorphometry				Dynamic histomorphometry	
		Day 0 (n = 9)	Day 60 (n = 9)	Day 60R (n = 4)		Day 0 (n = 9)	Day 60 (n = 9)	Day 60R (n = 9)		Day 60 (*n* = 9)
	BV/TV (%)	29.4 ± 5.0	32.0 ± 6.4	35.7 ± 17.1	BV/TV (%)	29.6 ± 10.9	24.7 ± 10.6	33.0 ± 15.7	sLS/BS (%)	26.2 ± 5.4
	TbTh (mm)	0.16 ± 0.02	0.17 ± 0.02	0.20 ± 0.07	ES/BS (%)	1.96 ± 2.55^a^	0.19 ± 0.17^b^	0.12 ± 0.05^a^	dLS/BS (%)	4.21 ± 2.17
	TbN (/mm)	1.73 ± 0.22	1.72 ± 0.28	1.9 ± 0.4	NOb/TA (/mm^2^)	75.9 ± 58.1	63.6 ± 27.0^c^	72.5 ± 22.1	MAR (μm/d)	1.16 ± 0.23
	ConnD (/mm^3^)	7.84 ± 1.88	7.90 ± 2.79	9.1 ± 3.4	NOc/TA (/mm^2^)	0.45 ± 0.38	0.01 ± 0.02	1.00 ± 0.75	BFR/TV (%/yr)	26.2 ± 7.4
Tiludronate	MicroCT				Static Histo				Dynamic Histo	
		Day 0 (n = 5)	Day 60 (n = 5)			Day 0 (n = 5)	Day 60 (n = 5)			Day 60 (n = 5)
	BV/TV (%)	28.1 ± 3.9	31.1 ± 5.0		BV/TV (%)	24.1 ± 7.2	30.8 ± 6.8		sLS/BS (%)	24.4 ± 7.0
	TbTh (mm)	0.16 ± 0.01	0.16 ± 0.02		ES/BS (%)	2.86 ± 1.38	1.49 ± 0.65^b^		dLS/BS (%)	6.33 ± 4.59
	TbN (/mm)	1.65 ± 0.17	1.81 ± 0.24		NOb/TA (/mm^2^)	29.7 ± 17.6	17.8 ± 11.3^c^		MAR (μm/d)	1.16 ± 0.21
	ConnD (/mm^3^)	7.46 ± 1.28	9.39 ± 3.17		NOc/TA (/mm^2^)	0.61 ± 0.41	0.06 ± 0.11		BFR/TV (%/yr)	29.8 ± 12.6
Clodronate	MicroCT				Static Histo				Dynamic Histo	
		Day 0 (n= 3)	Day 60 (n = 5)	Day 60R (n = 5)		Day 0 (n = 5)	Day 60 (n = 5)	Day 60R (n = 5)		Day 60 (n = 5)
	BV/TV (%)	32.3 ± 3.6	31.7 ± 2.9	39.2 ± 10.6	BV/TV (%)	30.7 ± 3.9	23.4 ± 4.8	34.5 ± 9.4	sLS/BS (%)	28.4 ± 19.0
	TbTh (mm)	0.15 ± 0.02	0.16 ± 0.01	0.20 ± 0.04	ES/BS (%)	0.02 ± 0.03^d^	0.14 ± 0.13	0.13 ± 0.06^d^	dLS/BS (%)	4.3 ± 4.6
	TbN (/mm)	1.95 ± 0.11	1.87 ± 0.18	2.22 ± 0.25	NOb/TA (/mm^2^)	88.3 ± 32.5	49.6 ± 16.0	63.4 ± 17.6	MAR (μm/d)	1.02 ± 0.29
	ConnD (/mm^3^)	9.75 ± 1.02	8.85 ± 1.80	13.50 ± 2.96	NOc/TA (/mm^2^)	0.44 ± 0.61	0.04 ± 0.05	0.84 ± 0.40	BFR/TV (%/yr)	25.5 ± 15.2

Abbreviations: BV/TV, bone volume per total volume; TbTh, trabecular thickness; TbN trabecular number; ConnD, connectivity density; ES/BS, eroded surface per bone surface; NOb/TA, number of osteoblasts per tissue area; NOc/TA, number of osteoclasts per tissue area; sLS/BS, single label surface per bone surface; dLS/BS, double label surface per bone surface; MAR, mineral apposition rate; BFR/TV, bone formation rate per total volume

Superscripts designate a significant difference of *P* < 0.05 between the same letters. Summary of bone analyses for treatment groups

### Histology

**Fig. 2:**
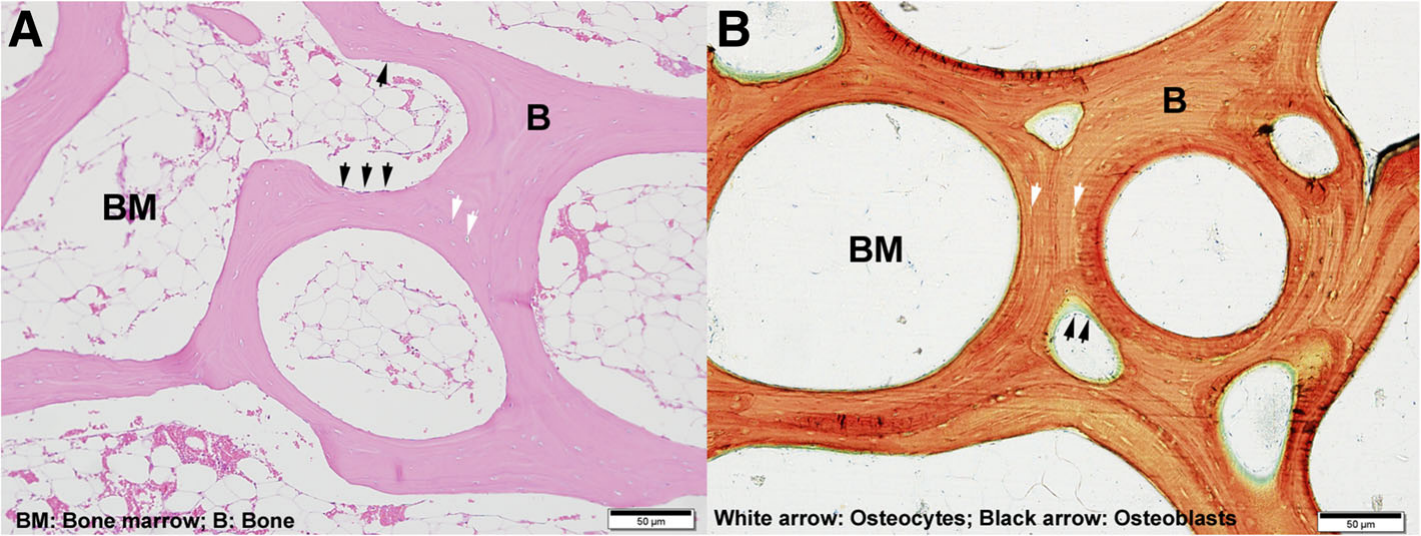
Overview of bony features for different histological preparations. Representative histologic sections of (**a**) decalcified Day 0 biopsy and (**b**) un-decalcified Day 60 biopsy, highlighting similar trabecular bone features. Abbreviations: B, bone; BM, bone marrow. Black arrows = osteoblasts; white arrows = osteocytes

Following microCT evaluation, biopsies were prepared for histology. Day 0 and Day 60R biopsies were prepared for decalcified histology, and Day 60 biopsies were prepared for plastic-embedded undecalcified histology using standard methods so visualization of fluorochrome labels was possible. Due to differences in preparation techniques between decalcified and undecalcified histology, and size of slides used for each, histological preparation of both sets of biopsies differed slightly. However, care was taken to ensure sections from each biopsy were taken in the same location and plane of section, regardless of preparation method. Day 0 and Day 60R biopsies were decalcified with 10% ethylenediaminetetraacetic acid (EDTA) and prepared for routine paraffin embedding. In order to fit on glass histological slides, Day 0 biopsies were split in half and embedded separately. This resulted in 6–8, 4 μm thick sections per biopsy that were stained with hematoxylin and eosin (H&E). Day 60 biopsies were prepared intact for undecalcified histology and two, 45–60 μm thick sections were produced. The first was stained with Stevenel’s Blue and Van Gieson’s picrofuchsin for evaluation by static histomorphometry, and the second section was left unstained for evaluation by dynamic histomorphometry. Although histological slides for Day 0 and Day 60 biopsies were prepared differently for ease of handling, all bony features were easily discernable and comparable [25, 26] in both sets of slides, as outlined in Fig. 2.

All bone histomorphometry evaluations were performed by an individual (HAR) using histomorphometry software (Osteomeasure© Bone Histomorphometry System, Osteometrics, Inc., Decatur, GA, USA) following standard procedures and nomenclature [26]. Measurements were taken per standardized and published protocols [26]. Static histomorphometry parameters included direct measurements of: tissue area (TA), BV, marrow volume (MaV), bone perimeter (BPm), osteoblast perimeter (ObPm), bone surface (BS), eroded surface (ES), reversal surface (RevS), osteoblast number (NOb), osteoblast surface (ObS), osteoclast number (NOc), osteoclast surface (OcS). Indirect measurements included: TbN, TbTh, TbSp, and the aforementioned direct measurements standardized to respective areas and volumes. Osteoblasts and osteoclasts were identified by their unique morphologic characteristics [27, 28]. A detailed list of these parameters, including definitions, has been previously published by Dempster, et al. (Tables 3 & 5 within reference [26]). However, due to normal variation in biopsy sizes, only parameters normalized to areas and volumes were evaluated statistically. Dynamic histomorphometry included mineralizing surface per bone surface (MS/BS), mineral apposition rate (MAR) and the bone formation rate per total volume (BFR/TV). A detailed list of these parameters, including definitions, has been previously published by Dempster, et al (Tables 4 & 5 within reference [26]).

### Statistics

Data were tested for normality using the Shapiro-Wilk test, and depending on normality, either parametric or non-parametric analyses were used. If data were parametric, microCT and histomorphometry data were analyzed using one-way ANOVA analysis to determine the effect of treatment within Day 0 or Day 60 biopsy. If significance was found, a Tukey post-hoc analysis was evaluated. If data were nonparametric, microCT and histomorphometry data was analyzed using an Independent Samples Mann-Whitney U Test, comparing TIL or CLO to SAL. A Student’s t-test was performed to determine the effect of clodronate on bone healing parameters, comparing CLO to SAL within Day 60R biopsy. Additionally, a related-sample test was used to evaluate changes from Day 0 biopsy to Day 60R biopsy, within the SAL or CLO horses. Statistical analyses were performed using commercially-available software (SPSS, version 22, IBM Corporation, Armonk, NY, USA), and *p*-values of < 0.05 were considered significant. Due to the aforementioned issues with obtaining microCT data from two Day 0 biopsies, all data from those horses were excluded from dependent analyses.

## Results

### Surgical technique

All surgeries were successful in obtaining adequately sized bone biopsies during each attempt, with only one horse experiencing incisional dehiscence. However, this complication resolved with minimal medical intervention. No horses displayed any rear limb lameness or ratable discomfort at any time over the duration of this study. Day 0, Day 60 and Day 60R biopsies had a mean (± SD) volume of 1632 ± 785, 1347 ± 622 and 1080 ± 646 mm^3^, respectively.

### Bone morphology

Select results are outlined in Table 2. When comparing microCT data between three treatment (saline, clodronate, tiludronate) groups within Day 0 biopsy or within Day 60 biopsy, there was no significant effect of treatment on any of the 19 bone morphology parameters as evaluated by microCT. Similarly, there were no significant changes in bone morphology parameters between Day 0 and Day 60 biopsies within treated horses as evaluated by microCT. However, when evaluating bone morphology using static histomorphometry, TIL-treated horses had a significant increase in BS/TV after 60 days when compared to control (*p* = 0.02).

### Cellular structure

Select results are outlined in Table 2. There were minor differences when evaluating bone formation parameters. When comparing TIL-treated horses to control horses within the Day 60 biopsy, there was a significant decrease in NOb/TA (*p* = 0.004) and NOb/BPm (*p* = 0.003), and a significant increase in NOb/ObPm (*p* < 0.000). When making the similar comparison within Day 60 biopsy, CLO-treated horses did not have significantly different bone formation parameters when compared to control. Overall, TIL significantly altered three of four normalized bone formation parameters, while CLO did not have an effect.

There were also minor differences when evaluating bone resorption parameters. When comparing TIL-treated horses to control horses, there was a significant increase in ES/BS (*p* = 0.001) and RevS/BS (p = 0.001) after 60 days. There were no significant changes in bone resorption parameters within CLO-treated horses compared to control horses after 60 days. Overall, TIL significantly altered two of six normalized bone resorption parameters, while CLO did not have an effect.

### Bone remodeling kinetics

Select results are outlined in Table 2. After 60 days, there was no effect of either treatment on any of the 14 bone remodeling parameters evaluated via dynamic histomorphometry.

### Bone healing

When comparing CLO-treated horses to control horses within the Day 60R biopsy, there was no significant effect on any of the 19 bone morphology parameters, as evaluated by microCT (Table 2). When comparing changes from baseline biopsy to Day 60R biopsy within CLO-treated and SAL-treated horses, there were no significant differences in any of the 19 bone morphology parameters evaluated via microCT. Comparing CLO-treated horses to control horses within the Day 60R biopsy using histomorphometry, treated horses had significantly higher BS/TV (*p* = 0.02) and TbN (p = 0.02). However, as outlined above, these results were not confirmed with microCT analyses, and overall, this accounts for two out of 20 bone morphology parameters that were significantly affected.

When evaluating changes from baseline to Day 60R within CLO-treated horses, there was significantly higher ES/BS (*p* = 0.03), as evaluated by static histomorphometry. However, the same effect was seen in the control horses after 60 days (ES/BS; *p* = 0.049). Comparing CLO-treated horses to control horses within the Day 60R biopsy, CLO-treated horses had significantly less ObS/BS (p = 0.04). Overall, this accounts for one of four bone formation parameters, with no bone resorption parameters affected.

**Fig. 3:**
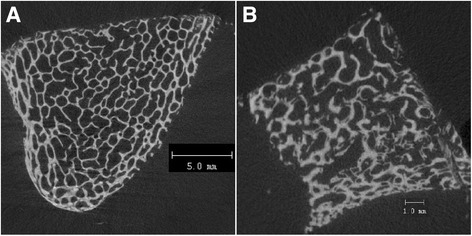
MicroCT slices demonstrating morphological evidence of trabecular remodeling. Two dimensional microCT slices representing a 30 μm thick section of an original biopsy (**a**) and re-biopsy (**b**) from the tuber coxae. Evidence of reconstitution and maturation of the trabecular structure is evident in (**b**), indicating active bone remodeling following the original biopsy

While few significant changes were identified in bone morphology parameters following re-biopsy in the control horses, there were areas identified within the re-biopsy samples that clearly demonstrated reconstitution and maturation of trabecular structure (Fig. 3). This indicates that the Day 60R samples were composed at least in part of non-native bone.

## Discussion

This report describes the effect of a single administration, consistent with clinical dosages and administration, of tiludronate or clodronate on bony cells, trabecular bone morphology, bone remodeling, and bone regeneration in ten healthy, young horses. A previous study utilizing a bone biopsy model attempted to elucidate changes in bone morphology secondary to tiludronate treatment in horses [19]. Unfortunately, the samples obtained in that study were inadequate for evaluation of bone morphology and remodeling; therefore, the current study provides these missing data, and includes preliminary data demonstrating no effect on bone regeneration following re-biopsy of the original biopsy site. Additionally, there have been no studies attempting to evaluate the effect of clodronate, despite its prolific use in veterinary clinics. A barrier to the veterinary field is that there is currently no data regarding whether tiludronate or clodronate affects bone remodeling in the horse at the recommended dosage and route of administration. Therefore, the current study provides these preliminary data indicating that there may be little to no effect on bone remodeling at this timepoint. To our knowledge, this is also the first study evaluating the cellular and structural effect of tiludronate and clodronate in horses of any age, as no published studies have evaluated these changes in either skeletally mature or immature horses. There were no significant effects of tiludronate or clodronate on bony cells or bone structure and remodeling parameters after 60 days in young horses, nor any significant differences in treatment groups following induction of an injury (re-biopsy of the original biopsy site). Treatment success in the previous study [1] may have been based on clodronate’s pain-minimizing properties, and not due to changes in bone remodeling. This would explain the treatment success demonstrated in that clinical trial [1], but support the lack of bony changes shown in this study.

Overall, there were no effects on bone resorption or bone remodeling kinetics, and only slight alterations in bone formation parameters. Bisphosphonates, in general, are widely known to affect bone remodeling kinetics, specifically suppressing bone remodeling, as measured by dynamic histomorphometry using fluorochrome labeling [29]. In the present study, there were bone morphology changes found during static histomorphometric evaluations that were not confirmed in microCT results. Therefore, they were not considered in overall conclusions, as microCT is a 3D measurement that accounts for the entire biopsy, and has greater sensitivity to bony measurements [24] when compared to histomorphometry. Additionally, when considering only a couple of bone formation parameters were affected out of over 20 parameters evaluated, including both directly measured and calculated, the minor changes to bone formation in the TIL-treated group is not considered a significant conclusion.

A limitation of the current study is a low sample size of 5 horses per group; therefore, it is possible that a small effect of tiludronate or clodronate on bone remodeling and structure may not have been apparent, or that the minor changes in bone formation parameters may indicate true changes that would be more apparent using a larger study population. However, it is well known that bisphosphonates are powerful modifiers of the bone remodeling process, including in normal bone [30]. Therefore, it was hypothesized that if there were a systemic effect of tiludronate or clodronate administration in the horse at the routine clinical dose administered, it would have been detected in the current study. In addition, a recent study evaluating the effect of the bone resorption inhibitor cathepsin K, which does not fully prevent osteoclastic resorption as bisphosphonates have been shown to do [31], was performed with groups of 6 horses, with significant differences found [32]. Therefore, we conclude that the lack of changes identified in the current study, especially related to the re-biopsy results and effects on a model of bone healing, are truly reflective of an overall lack of influence of these drugs on bone remodeling kinetics in the horse. The authors are currently unsure of why a lack of effect was found in these studies, and if horses have a different response to these drugs than other species. Ongoing work is being conducted to evaluate the effectiveness of these drugs to bind to equine bone and the effect on the tissue and bony cells in vitro. In addition, we are exploring whether more modern bisphosphonates and/or other drugs used to modify bone remodeling in other species have an effect in the horse.

It is possible that the time frame (60 days) evaluated in this study was not appropriate to evaluate overall changes in bone remodeling. As outlined in the methods, this time frame was selected based on the only published data regarding the effect of tiludronate and clodronate on horses, which found significant clinical improvements in horses diagnosed with navicular disease, distal tarsal osteoarthritis and osteoarthritis in the axial skeleton after 60 days [1, 2, 5, 6, 21]. In addition, a study evaluating bone remodeling in horses following treatment with phenylbutazone, which, unlike tiludronate, is not utilized as a treatment for diseases of bone remodeling, found significant changes in MAR using similar sample sizes after only 30 days [33]. Therefore, it would be expected that normal bone apposition rates in horses should be affected by tiludronate and clodronate within the study timeframe. However, future studies including additional time points post-treatment would be required to confirm whether tiludronate or clodronate has a significant impact on bone morphology. Similar work would need to be done to fully characterize intramembranous and/or endochondral bone regeneration and remodeling process following injury (i.e., re-biopsy) at this location in order to determine an optimal time point to evaluate potential effects of these drugs on bone regeneration in the horse. As such, more recent data evaluating effectiveness of tiludronate administration on lameness associated with navicular disease as evaluated by more objective force platform analyses found significant improvements 120 and 200 days post treatment, but not after 60 days [4]. Ideally, the studies presented herein would be based on known timeframes regarding bone remodeling kinetics in the horse as opposed to strictly clinical outcomes data; however, the authors are not aware such data exists.

Tiludronate and clodronate therapies are widely used in the clinical setting to treat disorders involving abnormal bone remodeling, including distal tarsal joint osteoarthritis [6], thoracic & lumbar vertebral arthritis [21], and dorsal metacarpal disease [34]. Evaluations of tiludronate on skeletally immature rats and baboons has been reported [35, 36], and showed a reduction of trabecular bone resorption adjacent to growth plates and increased bone density. Tiludronate and clodronate have been previously evaluated for pathological bone changes in horses aged 4 years and older [1, 2]; however, clinicians are administrating these drugs to horses that are younger than 4 years of age, including off-label use for treatment of dorsal metacarpal disease [34]. It is well known that younger horses undergo more rapid bone remodeling than skeletally mature adults [37], and these differences would be reflected in the chosen biopsy site, so this study aimed to also evaluate the effect of tiludronate and clodronate administration in young horses, where negative impacts on normal bone remodeling could result in detrimental clinical outcomes in young equine athletes. As outlined above, we saw minimal, if any, influence on bone remodeling or morphology in this young population, and we would hypothesize to see similar results in skeletally mature horses. However, due to the smaller sample size for each age included the study and the known effect of age on bone remodeling parameters, there cannot be definitive conclusions based on age. In addition to a larger number of subjects, a smaller range of ages in subjects may have resulted in a significant effect in other bone morphology parameters.

The iliac biopsy location utilized in the study was chosen for multiple reasons. First, it has been shown that bisphosphonates accumulate at the highest density in the tuber coxae when compared to the 3rd metacarpal, rib, and fourth tarsal bone in the horse [19]. Secondly, it is an easily accessible location that allowed for a non-terminal model. Thirdly, it has been shown that the tuber coxae accurately predicts the effect of alendronate, another form of bisphosphonate, on non-homogenous skeletal locations in the body (i.e., vertebral column) [38]. Bisphosphonates have been repeatedly shown to have systemic effects on bone [39], especially trabecular bone [40], so it can be reasonably argued that any changes that would occur secondary to treatment with clodronate would be identified in the selected biopsy location. The anatomical location of the biopsy does have a few limitations, including the lack of compressive forces upon it as one would see in bony tissue of the distal limb; however, the benefits of this biopsy method (e.g., ease of obtaining a sample, large size, presence of both trabecular and cortical bone) far outweigh the negatives [17]. However, care must also be taken when evaluating the re-biopsy results, as this model is novel and the exact timeline of intramembranous and/or endochondral ossification at this location in the horse has yet to be explored in detail. The morphology of the samples (Fig. 3) indicates that at least part of the re-biopsies contained non-native bone and therefore would have been subjected to extensive remodeling during the study timeframe. Given the relatively large biopsy size, this study was able to utilize multiple study endpoints, including both static and dynamic histomorphometry. The second biopsy was prepared for un-decalcified histology in order to preserve the fluorescent label, which required different preparation than the initial biopsy received. Although preparation methods varied, methods of analyses and outcome measures were identical for both preparation methods of static histomorphometry sections [26]. In addition, recent work has identified that outcomes of evaluations of bony tissue and cells do not differ between the two preparation techniques [25].

## Conclusions

In conclusion, this study utilized several established methods to evaluate bone morphology and remodeling secondary to treatment with either tiludronate or clodronate from a non-terminal bone biopsy method of the equine tuber coxae. These data demonstrate that tiludronate and clodronate do not have a substantial impact on normal bone remodeling kinetics, morphology or bony cells in young horses 60 days post-treatment. With the lack of alteration of any expected bone remodeling parameters in these horses, the authors are unsure if clinical improvement in lameness is due to effects on bone remodeling or the analgesic effect of clodronate or other effects secondary to bisphosphonate administration in the horse. More studies are warranted to further explore these negative findings, including the investigation of clinically diseased horses.

## Abbreviations

AAEP: American Association of Equine Practitioner
BFR: Bone formation rate
BPm: Bone perimeter
BS: Bone surface
BV: Bone volume
CLO: Clodronate
ConnD: Connectivity density
dLS: Double label surface
EDTA: Ethylenediaminetetraacetic acid
ER: Eroded surface
FOI: Freedom of Information
H&E: Hematoxylin and eosin
IACUC: Institutional Animal Care and Use Committee
IM: Intramuscular
IV: Intraveneous
MAR: Mineral apposition rate
MaV: Marrow volume
microCT: Micro-computed tomography
NOb: Osteoblast number
NOc: Osteoclast number
ObPm: Osteoblast perimeter
ObS: Osteoblast surface
OcS: Osteoclast surface
RevS: Reversal surface
SAL: Saline
sLS: Single label surface
SMI: Structural model index
TA: Tissue area
TbN: Trabecular number
TbSp: Trabecular separation
TbTh: Trabecular thickness
TIL: Tiludronate
TV: Total volume
